# Microscopic Structure of Liquid Nitric Oxide

**DOI:** 10.1021/acs.jpcb.2c05384

**Published:** 2022-11-18

**Authors:** Sarantos Marinakis, Cillian Cockrell, Kostya Trachenko, Thomas F. Headen, Alan K. Soper

**Affiliations:** † Department of Chemistry, University of Patras, PatrasGR-26504, Greece; ‡ School of Physics and Astronomy, Queen Mary University of London, Mile End Road, LondonE1 4NS, U.K.; § ISIS Facility, STFC-UKRI Rutherford Appleton Laboratory, Harwell Campus, Didcot, OxonOX11 0QX, U.K.; ∥ School of Health, Sport and Bioscience, University of East London, Stratford Campus, Water Lane, LondonE15 4LZ, U.K.

## Abstract

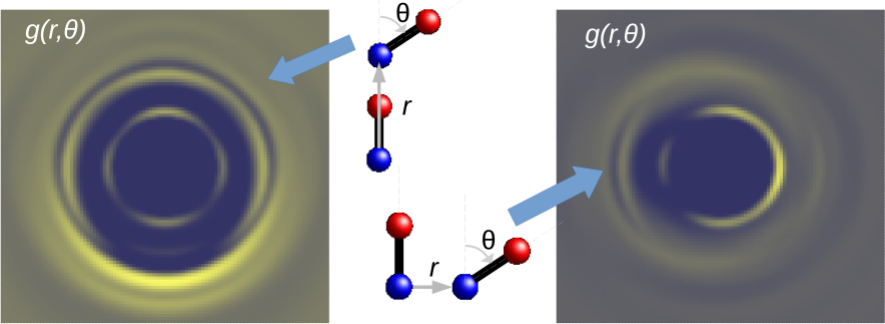

The microscopic structure of nitric oxide is investigated using neutron scattering experiments. The measurements are performed at various temperatures between 120 and 144 K and at pressures between 1.1 and 9 bar. Using the technique of empirical potential structure refinement (EPSR), our results show that the dimer is the main form, around 80%, of nitric oxide in the liquid phase at 120 K, but the degree of dissociation to monomers increases with increasing temperature. The reported degree of dissociation of dimers, and its trend with increasing temperature, is consistent with earlier measurements and studies. It is also shown that nonplanar dimers are not inconsistent with the diffraction data and that the possibility of nitric oxide molecules forming longer oligomers, consisting of bonded nitrogen atoms along the backbone, cannot be ruled out in the liquid. A molecular dynamics simulation is used to compare the present EPSR simulations with an earlier proposed intermolecular potential for the liquid.

## 1 Introduction

Nitric oxide (NO) is a ubiquitous signaling molecule in the cardiovascular system and plays a significant role in vasodilation.^1^ NO along with nitrogen dioxide (NO_2_) is the main product in combustion chemistry, and thus it is one of the most important pollutants in the urban atmosphere.^2^ Despite its significance, the physicochemical properties of nitric oxide are not well understood. This is not only because it is a toxic and oxidizing agent to many other chemicals but also because it is prone to make dimers, trimers,^3^ and oligomers with itself.^4^ The extent of this clustering is presumably dependent on the temperature and pressure, but it is not yet fully characterized. The clustering of NO on surfaces is a very active field of research (see refs (5−14) and references therein). The existence of the dimer is predominant in the liquid phase but not in the gas phase.^15^ Moreover, according to the same citation, the proportion of dimers in the liquid declines rapidly as the critical point is approached. Exact calculations have not been done yet because there are 16 electronic states involved.^16^ A detailed understanding of the microscopic structure and dynamics will be useful in a rich variety of systems including biochemistry processes, in exact calculations of NO_*x*_ species in high-pressure combustion chemistry, and in understanding and improving the use of radicals in solutions. In its liquid form NO can explode if it is exposed to a sudden force or concussion.^17^

The nitric oxide molecule has attracted many theoretical and experimental studies. On the theoretical side, the NO dimer has been extensively studied using various density functional theory (DFT) and *ab initio* methods. We refer the reader to the most recent and accurate calculations (refs (3 and 18−20) and references therein). DFT methods have so far not been successful in deriving the proper geometry and relative energy of the cis- and trans-NO dimers.^18^ Sayós et al.^18^ used multiconfigurational second-order perturbation theory (CASPT2) to obtain shallow (NO)_2_ with singlet and triplet electronic configurations for both the cis- and trans-NO dimer. The most stable was the ^1^A_1_ cis-NO state. The authors also studied the nonplanar transition state for the singlet trans–cis isomerization which had an almost negligible energy barrier. Marouani et al.^19^ performed multireference configuration interaction (MRCI) calculations with large diffuse basis set to get the potential curves and the spin–orbit couplings. Ivanic et al.^20^ used multireference second-order perturbation theory (MRMP2) and complete basis set (CBS) limit extrapolation to investigate (NO)_2_ and (NO)_4_. By using their theoretical predictions for the vibrational frequencies, they suggested that (NO)_4_ had been observed in previous experiments. Hoshina et al.^3^ used MRMP2/CBS calculations for the dimer and MRMP2 level of theory with quadruple-ζ (QZ) basis set to study the trimer and tetramer to compare with infrared experiments of NO clusters in He droplets. Their calculations showed that the trimer was stable relative to NO + (NO)_2_ by *D*_e_ = 529 cm^–1^, and the tetramer was stable relative to separation into two dimers by *D*_e_ = 889 cm^–1^.

A number of experimental and theoretical studies have alluded to the fact that in the liquid state near the melting point the liquid occurs primarily in the form of dimers, (NO)_2_, but as the temperature is raised along the coexistence curve, these dimers increasingly dissociate to the monomeric form, NO. On the assumption that the monomer is paramagnetic, while the dimer has very low or zero magnetic susceptibility, Smith and Johnston^21^ used measurements in the coexistence region between 110 and 120 K to establish that the degree of dissociation of NO liquid is in the region of 0.027–0.051, corresponding to mole fractions of (NO)_2_ ((1 – α)/(1 + α), α is the degree of dissociation) of 0.95–0.91. Subsequent theoretical studies^15,22−25^ suggest that the mole fraction of (NO)_2_ in the liquid has to decrease with increasing temperature, reaching close to zero near and above the liquid vapor critical point.

On the structural side, the structure of the dimer in the gas phase was studied using high-resolution microwave (MW) and radio-frequency spectroscopy in 1981 and was found to have a symmetric cis-planar ONNO structure.^26^ In the solid phase, previous experimental work (X-ray) was published in 1951 and 1953,^27,28^ where the proposed structure of the dimer at a temperature of 109 K was nearly rectangular. However, the results of that work were reinterpreted again in 1961, and a trapezoidal structure of *C*_2*v*_ symmetry was proposed.^29^ The more recent study, in 1989, of the microscopic structure of ^14^NO and ^15^NO was on the liquid state.^30^ The experiment was performed at the ISIS pulsed neutron source at a temperature of 120 K. That work suggested that most of the nitric oxide exists as a cis-planar dimer. Finally, there is an open debate and striking disagreement between the experimental work in the liquid phase^30^ and more recent reverse Monte Carlo (RMC) simulations,^31^ which were based on the same diffraction data as ref (30). The RMC simulations appeared to show that the experimental data were not consistent with a cis-planar model of the dimer. The authors doubted not only the dominance of the cis-planar dimers but also the existence of them at all in the liquid phase. They proposed that alternative models, such as T-configuration and “parallel” configurations, are very probable. Our study aims to try to answer to this debate.

There are many good reasons to perform more work on such an important system. First, there were some difficulties with structural experiments in the liquid state. As the authors mentioned,^30^ hafnium was present as an impurity in the titanium–zirconium alloy used for the sample containment, and this caused problems with the data in the low-wavelength regions. We were able to remedy this problem by using modern TiZr alloys which contain no Hf. Second, the published results so far on the microscopic structure of the dimer of NO were obtained only at three thermodynamic points: 109 K in the solid phase, 120 K and 1.1 bar in the liquid phase, and room temperature in the gas phase. The new measurements at various thermodynamic points along with the accompanying calculations will test the existence and dominance of various models of the dimer in a much wider range in the *pVT* diagram.

## 2 Methods

### 2.1 Experimental Section

A cylinder of nitric oxide was obtained from CK Special Gases (99%) and used without further purification. The pressure of the cylinder was around 18 bar, and a regulator connected to a manifold system was used to reduce the pressure. Capillaries were used to connect the manifold system with the cell. The flat plate pressure cell was made from an alloy of Ti and Zr in the mole ratio 0.676:0.324, which contributes almost zero coherent scattering to the diffraction pattern.^32^ The cell consisted of a flat section that had 2 mm path length and 1 mm wall thickness. The height and width of the cell were larger than the neutron beam size.

The container was placed at a right angle to the neutron beam, which was set to 30 mm × 30 mm using six sets of adjustable collimating jaws between the moderator and sample position. A top loading closed cycle helium refrigerator (CCR) using He exchange gas at 20 mbar to provide temperature uniformity was used to reduce the temperature with fine control provided by heaters at the top and bottom of the cell. Typically, the CCR temperature was set 10 K below the cell temperature. The temperature was controlled within ±0.2 K. Nitric oxide has a low freezing (109.51 K) and boiling temperature (121.38 K), and its critical point (*T*_c_ = 180.00 K, *P*_c_ = 64.80 bar, *V*_c_ = 58.00 cm^3^/mol) allows its use at ambient pressure.^33^

The employed temperatures and pressures are shown in Table 1 and were selected near the vapor–liquid saturation line using data from REFPROP.^34^ To make sure that the neutron beam was hitting only liquid, we used slightly higher pressures than the vapor–liquid saturation curve.

**Table 1:** *T*–*P*–*d* State Points for Neutron Scattering Measurements^a^

*T*_exp_ [K]	*P*_exp_ [MPa]	*d*[kg/m^3^]
120.0	0.133	1273.7
127.0	0.208	1239.3
134.0	0.409	1199.5
144.0	0.890	1129.5

a The values of *d* are taken from ref (34).

**Figure 1:**
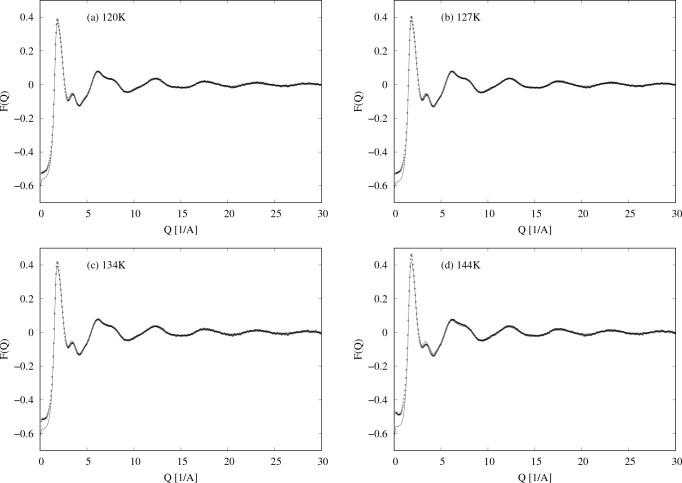
Four neutron diffraction data sets for NO at (a) 120, (b) 127, (c) 134, and (d) 144 K. The dots are the diffraction data, and the lines are EPSR fits to the data using only NO monomers as described in Section 3.2.

Total neutron scattering measurements were performed on the NIMROD diffractometer at the ISIS pulsed neutron source.^35^ Absolute values of the differential cross sections were obtained from the raw scattering data by normalizing the data to the scattering from a 3 mm slab of nonscattering vanadium–niobium alloy 0.9485:0.0515, which has a known scattering cross section and density, and were further corrected for background and multiple scattering, container scattering, and self-attenuation, using the Gudrun data analysis program.^36^ Finally the data were put on absolute scale of barns per atom per sr by dividing by the number of atoms in the neutron beam (1 barn = 10^–28^ m^2^). The interference differential cross sections, *F*(*Q*), are shown in Figure 1.

### 2.2 Data Interpretation Using Empirical Potential Structure Refinement (EPSR)

The data shown in Figure 1 were subjected to a series of EPSRs to give an indication of the likely structural consequences of these data. The method is well-described in a number of references,^37−39^ and the details of these simulations are given in the Results section. The full set of computer files for these simulations are available for download at the website given at the end of this paper.

In essence, the EPSR method performs a constant volume Monte Carlo simulation of the system at the temperature and density in question, building in as much prior information (such as molecular structure, likely minimum atomic overlaps, likely atomic charges where relevant, and so on) as is available. Comparison of the differential cross sections derived from this simulation and the corresponding measured diffraction data is used because of the linear relationship (a Fourier transform) between the data in reciprocal (*Q*) space and the pair distribution functions in real (*r*) space, to derive an additional “empirical potential” which is refined continuously as the simulation proceeds and which is aimed to achieve the best possible agreement with the measured data. The quality of that agreement can then be used to assess the correctness, or otherwise, of the starting assumptions for the assumed structural parameters.

### 2.3 Data Interpretation Using Molecular Dynamics (MD)

MD simulations at the experimental temperatures and densities were performed on NO using the interatomic potentials of Lachet et al.^25^ This model represents the NO monomer as a single Lennard-Jones site, ϵ = 1.08088 kJ/mol (equivalent to 130 K, σ = 3.400 Å), and the NO dimer as two of these sites held apart at a fixed bond distance of 2.237 Å. The NO molecule therefore has no internal structure, according to this model, and does not experience electrostatic interactions. The ratio of monomers to dimers is fixed during each simulation and determined according to interpolation of data in ref (25).

The MD simulations were performed with the DLPOLY package.^40^ Systems of 2000 atoms in total were constructed at the target density and mole fraction. The cutoff, beyond which the interaction potential was neglected, in all systems was set at 10 Å. These systems were then equilibrated with a time step of 1 fs for 100 ps in the NVT ensemble using the Langevin thermostat with a 0.1 ps relaxation time. These equilibrium structures were then used as the initial conditions for the production runs, which were performed in the NVE ensemble for 1 ns at a time step of 1 fs. Structural data were collected from these NVE production runs for comparison with the EPSR data, as described in Section 4.3.

## 3 Results

**Figure 2:**
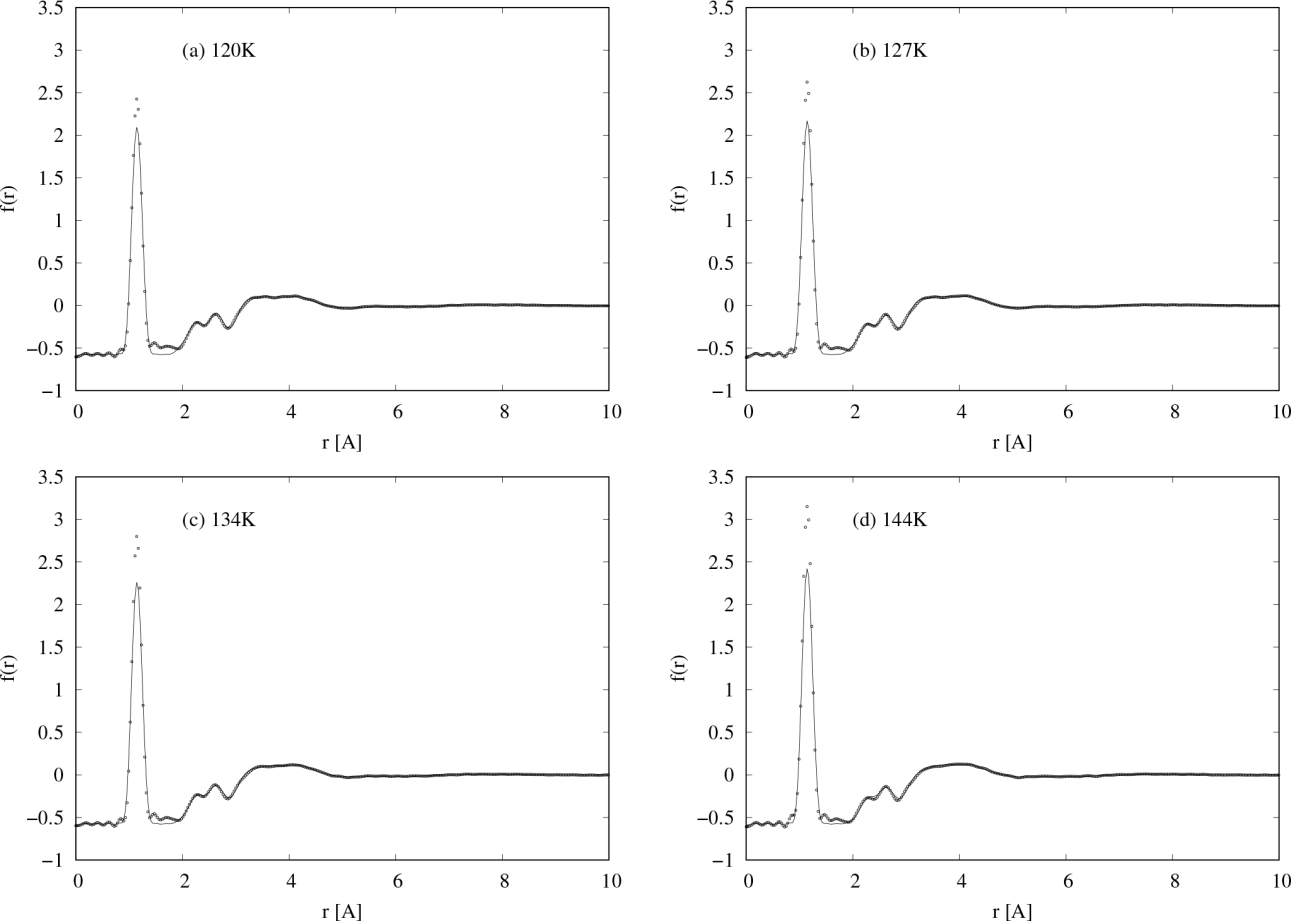
Total radial distribution functions for the *F*(*Q*) data shown in Figure 1. The dots are the diffraction data, and the lines are EPSR fits to the data using only NO monomers as described in Section 3.2.

When transformed to real space, *r*, the data of Figure 1 show a sharp peak at *r* = 1.15 Å, corresponding to the intramolecular NO bond, as seen in Figure 2. In addition, however, two other, much smaller, intramolecular peaks appear at *r* ∼ 2.25 Å and *r* ∼ 2.61 Å. Sharp peaks at these distances are uncommonly caused by typical intermolecular forces, such as overlap, dispersion, and so on, and the fact that there are two peaks suggests some form of orientational association between NO molecules, analogous to the phenomenon of hydrogen bonding between polar molecules containing hydrogen. This is precisely what Howe et al. concluded in 1989,^30^ although the real space resolution of their data was much poorer than the present data, and they were not able to resolve these two peaks separately, observing instead a single broader peak at *r* ∼ 2.4 Å.

It will be noted that in all these EPSR simulations the height of the first peak in *r*-space tends to be slightly underestimated, and there is an unaccounted for mismatch between simulation and data in the region 1.5–2 Å. Because there is unlikely to be genuine intensity from the liquid NO in this region, this discrepancy is believed to arise from difficult-to-remove residual background effects which arise when subtracting the large scattering from the empty container from the liquid plus container neutron scattering. In any case the presence of this discrepancy does not affect the primary conclusions made here from the data.

In fact, a number of X-ray diffraction and other studies converge to the conclusion that in the solid state NO occurs as the cis-planar O–N–N–O dimer.^27−29,41^ This was also the conclusion from analysis of the second virial coefficients by means of the principle of corresponding states,^42^ infrared-determined studies in the gaseous phase,^43,44^ UV–vis,^45^ molecular beam electric resonance (MBER) spectroscopy,^26^ microwave spectroscopy,^46^ matrix isolation experiments,^47^ far-infrared,^48^ and Raman^49^ as shown in Table 2. In fact, the existence of the trans-ONNO form was only proposed in matrix isolation experiments.^50,51^

**Table 2:** Geometrical Properties of (NO)_2_^a^

*r*_N–N_	*r*_N–O_	*∠*(NNO)	study
2.18(6)	1.12(4)	101(3)	X-ray (1971)^41^
2.33(12)	1.15(1)	95(5)	MBER (1981)^26^
2.236(1)	1.161(4)	99.6(2)	MW (1983)^52^
2.2630(12)	1.1515(3)	97.17(5)	FTIR (1995)^53^
2.278(31)	1.155(2)	97.8(6)	MW (1996)^54^

a Bond length and angles are in Å and deg, respectively. Indicated uncertainties in parentheses are 1σ in units of the last quoted digit of the parameter.

### 3.1 EPSR Modeling with a Mixture of Dimers and Monomers

**Figure 3:**
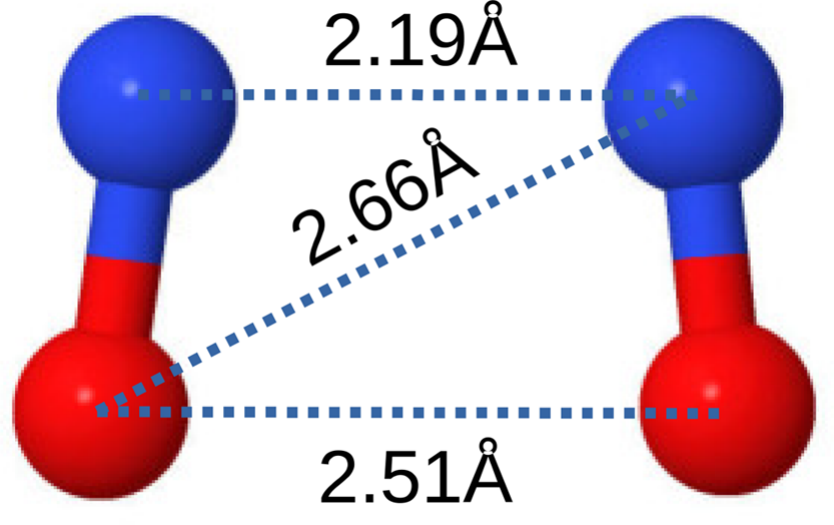
Picture of EPSR generated model of (NO)_2_ as a pair of bonded NO monomers forming a so-called “cis-planar” arrangement.^29,30^ Note the slightly trapezial shape of the molecule. Note that this is only one molecule out of many hundreds used in the simulations so that individual site–site distances may be different from the average values derived from the N–O distance and N–N–O angle listed in Section 3.1.

Using the technique of Empirical Potential Structure Refinement^37−39^ (EPSR), we built a model of the dimer (see Figure 3) that broadly satisfies previous measurements on the N–N distance and the N–N–O angle, as listed in Table 2. To obtain the best fit to the diffraction data, we used an average N–N–O angle of 94° and an N–N distance of 2.28 Å. The former value is slightly smaller than, but not inconsistent with, the previous measurements in the gas and solid phases, and the latter value is slightly larger (Table 2). The N–N correlation appears to be quite broad compared to the short-range N–O distribution which introduces a degree of uncertainty in assessing exactly what proportion of (NO)_2_ is present.

**Figure 4:**
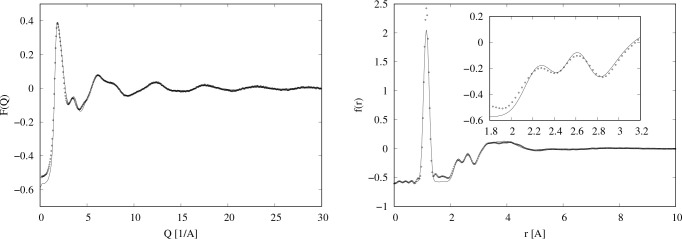
EPSR simulation without empirical potential refinement using a mixture ratio (NO)_2_:NO of 900:200 (mole fraction 0.818). The left figure is the fit in *Q*-space, while the right figure shows the fit in *r*-space. The inset shows the double peak at 2.1–2.85 Å, which indicates the presence of (NO)_2_ molecules in both the data and simulation.

To assess the relative amounts of (NO)_2_ and NO in the liquid, five simulated mixtures of the two molecules were prepared in the ratios 1000:0, 950:100, 900:200, 850:300, and 800:400, corresponding to the mole ratios for (NO)_2_ of 1.0, 0.905, 0.818, 0.739, and 0.667, respectively. These mole ratios were chosen as they spanned either side of the mole ratio which fit the data the best. The parameters of the (NO)_2_ molecule were adjusted to give best possible fits to the low *r* region, and the Lennard-Jones and charge parameters were set to those shown in Table 3. These mixtures were used in EPSR simulations *without* empirical potential refinement and produced a set of five simulations of the diffraction data, one for each mixture ratio. The simulation for the 900:200 mole ratio is shown in both *Q* and *r* spaces in Figure 4. The same discrepancy as seen in Figure 2 is seen here, which suggests it is not an artifact of the modeling regime.

**Table 3:** Lennard-Jones and Charge Parameters for (NO)_2_ and NO

atom	ε [kJ/mol]	σ [Å]	*q* [*e*]
N	0.124	3.31	–0.0286
O	0.204	2.95	+0.0286

**Figure 5:**
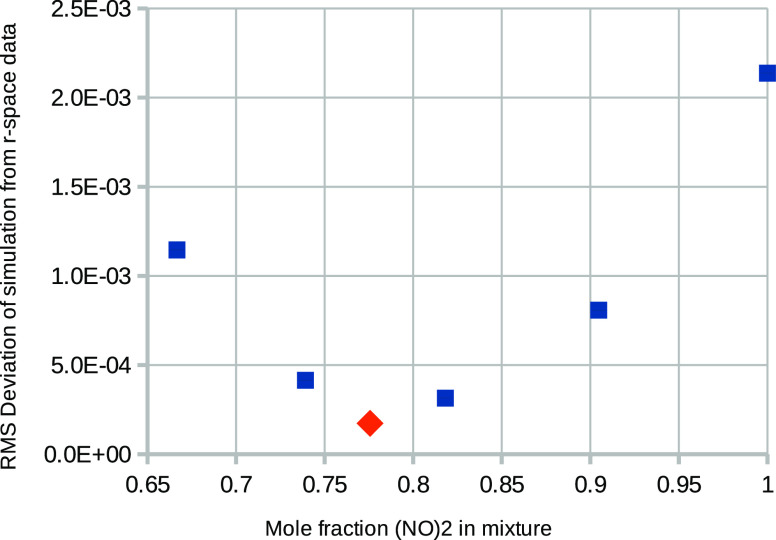
Quality of fit (root-mean-square deviation) of simulation to data in real space in the region *r* = 2.10 Å to *r* = 2.85 Å as a function of (NO)_2_ mole fraction for five mixture ratios (blue squares). The red diamond relates to the second method (described in Section 3.2) of determining the (NO)_2_ mole fraction from a simulation of purely monomers.

The quality of fit for these five mixture ratios is shown in Figure 5. Quality of fit is defined as the mean-square deviation between data and simulation in *r* space in the region between *r* = 2.10 Å and *r* = 2.85 Å, corresponding to the region where the extra N–N, N–O, and O–O intramolecular peaks from (NO)_2_ molecules occur. It can be seen that the best fits are obtained with mole ratios in the region of about 0.8 mole fraction of (NO)_2_.

### 3.2 EPSR Modeling with Only Monomers

In the method used above, the optimum ratio for the (NO)_2_ mole fraction is around 0.8 at 120 K. When attempting to fit this mole ratio to the higher temperatures of 127, 134, and 144 K, it became apparent that this was too high at the higher temperatures. In other words, there appears to be a decrease in this mole fraction of (NO)_2_ as the temperature increases. Using the above method of making a range of mixtures at each temperature, one could in principle determine the mole ratio at each temperature, but this is quite labor intensive because one needs to know the mole ratio *a priori*, and each simulation has to be equilibrated and then run for a long time to build up the required statistics.

To counter this drawback, an alternative approach has been adopted here in which we tried to force single NO molecules to form dimers of the kind shown in Figure 3, without specifying all the intramolecular geometry of the dimer. To do this, an alternative set of Lennard-Jones parameters was adopted to allow short-range N–N, N–O, and O–O intermolecular interactions between NO monomers. This basically involved weakening the potential significantly, as shown in Table 4.

**Table 4:** Weak Lennard-Jones and Charge Parameters Used to Generate (NO)_2_ Molecules in a Simulation Containing Only NO Molecules

atom	ε [kJ/mol]	σ [Å]	*q* [*e*]
N	0.010	2.90	–0.0286
O	0.010	2.99	+0.0286

**Figure 6:**
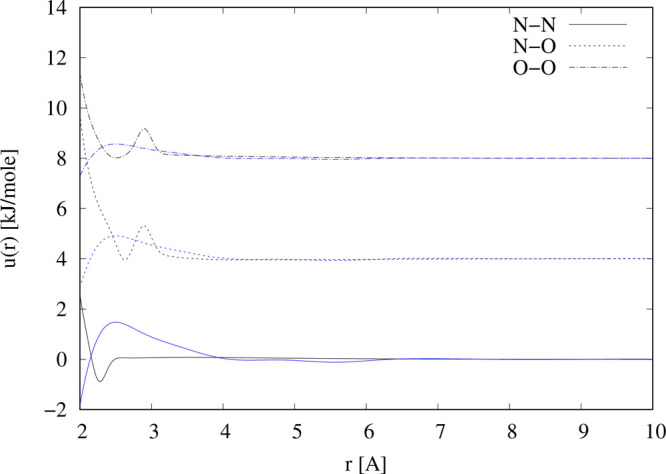
Intermolecular reference potentials (black) and empirical potentials (blue) used to simulate (NO)_2_ molecules from a box containing only NO molecules.

Additional Gaussian attractive potential terms of the form , where *A*_*i*_, *r*_*i*_, and σ_*i*_ are the amplitude (negative in the case of an attractive potential), position, and width, respectively, of the *i*th Gaussian potential, were combined with these Lennard-Jones potentials for the N–N, N–O, and O–O reference potentials at the positions 2.26, 2.61, and 2.44 Å, respectively, to emulate the expected intramolecular distances that would occur in the (NO)_2_ dimer. Further Gaussian repulsive terms (with positive amplitude) were added to the N–O and O–O reference potentials at 2.9 Å to demark the intramolecular interactions for the (NO)_2_ molecule from other intermolecular N–O and O–O interactions. The N–N coordination number associated with the peak at *r* = 2.24 Å was then controlled by a weak repulsive force that was added to the N–N reference potentials and set to give the best fit to this peak. The width and depth of the Gaussian terms were also determined by this requirement. Figure 6 shows the reference potentials that were obtained by these devices and detailed values of these potentials are given in the Supplementary Information.

It must be borne in mind that there is nothing particularly significant about these potentials—they are simply being used to obtain a satisfactory fit to the diffraction data—and it is likely other versions of these would have worked as well or better. They are designed to have the effect of creating (NO)_2_ molecules from a box containing only NO molecules. Figures 1 and 2 show the fits with these reference potentials in *Q* and *r* spaces, and in this case the empirical potential was allowed to increase to the extent necessary to get the best possible fits. The N–N coordination number in the range *r* = 1.80–2.52 Å  can be used to estimate the mole fraction of (NO)_2_ molecules. At 120 K the result is shown as the red diamond in Figure 5, with a mole fraction of about 0.78. This corresponds to the approximate value of 0.8 found in the first set of simulations with varying mole ratios of (NO)_2_:NO.

Furthermore, specifying short-range intermolecular interactions between NO monomers, in the way that is done here, in no way guarantees that *only* dimers can form, as occurs when (NO)_2_ molecules are specifically defined as in the previous section, nor that their conformation is as shown in Figure 3. Indeed, it is possible with this approach that longer chains of NO molecules might form and that they may have a range of conformations. In other words, this second approach allows a broader range of possible local NO structures to form and can help to identify which structures the diffraction data are sensitive to and which structures are not constrained by the data.

From these EPSR simulations, the variation of the N–N coordination number in the range *r* = 1.80–2.52 Å with temperature is shown in Table 5. It will be noted that if the liquid consisted only of pure (NO)_2_ dimers, this coordination number would be 1.0 exactly, whereas if the coordination number were zero, then the liquid would be made up only of monomers. Mixtures of monomers, dimers, trimers, and so on would give different N–N coordination numbers in this distance range. In practice, because these numbers are all <1.0, we will associate them here with the mole fraction of (NO)_2_ in the liquid, on the assumption that even if trimers, tetramers, and so on are present in the liquid to some extent, the dimer is the dominant nonmonomeric species.

**Table 5:** N–N Coordination in the Range *r* = 1.80–2.52 Å, Corresponding to the N–N Peak in the (NO)_2_ Dimer^a^

*T* [K]	N–N coordination number	assumed (NO)_2_ mole fraction
120	0.78	0.78
127	0.74	0.74
134	0.63	0.63
144	0.43	0.43

a In this work this coordination number is assumed equivalent the mole fraction of (NO)_2_ in the liquid.

## 4 Discussion

On the basis of the evidence presented in the previous section, there appears little doubt that in its liquid form near the ambient pressure boiling point, 120 K, nitric oxide occurs primarily as the dimer (NO)_2_ with mole fraction around 0.8, with the remaining NO molecules in monomeric form. The existence of the dimer in the solid state is well-known and predicted for the liquid in various *ab initio* calculations.^3,18−20,55,56^ As the liquid is heated to 144 K at pressures up to 9 bar the mole fraction of (NO)_2_ decreases to around 0.44, signaling behavior expected of a weak N–N interaction between pairs of NO molecules in the dimer.^56^

### 4.1 Presence of Other Structures besides Dimers

The model described in Section 3.1 makes a specific assumption about the planar structure of the dimer (Figure 3) and achieves an acceptable fit to the data (Figure 4) without the need for an added empirical potential. However, this by itself cannot be taken as evidence that *only* planar dimers occur in the real liquid, unless we can also demonstrate that models with nonplanar dimers, or trimers, or other structures are inconsistent with the data.

An exhaustive study of this question is beyond the scope of the present work, but an attempt to illustrate a possible answer is made in Section 3.2 where monomers are allowed to associate via a short N–N distance, with corresponding potential constraints on the N–O and O–O distances between neighboring NO molecules (Figure 6). Such a set of intermolecular potentials allows dimers of the specified kind to form but also may allow other possible structures to form which may not have been previously considered. In this case, we did allow the empirical potential to achieve the best possible fits at each temperature (Figures 1 and 2) because of the uncertainty of whether these additional potential constraints would be sufficient on their own to achieve a good fit to the data. In practice, these empirical potential contributions are small and featureless compared to the underlying reference potential in the region of the short-range NO–NO interactions, *r* = 2.0–2.9 Å (Figure 6).

**Figure 7:**
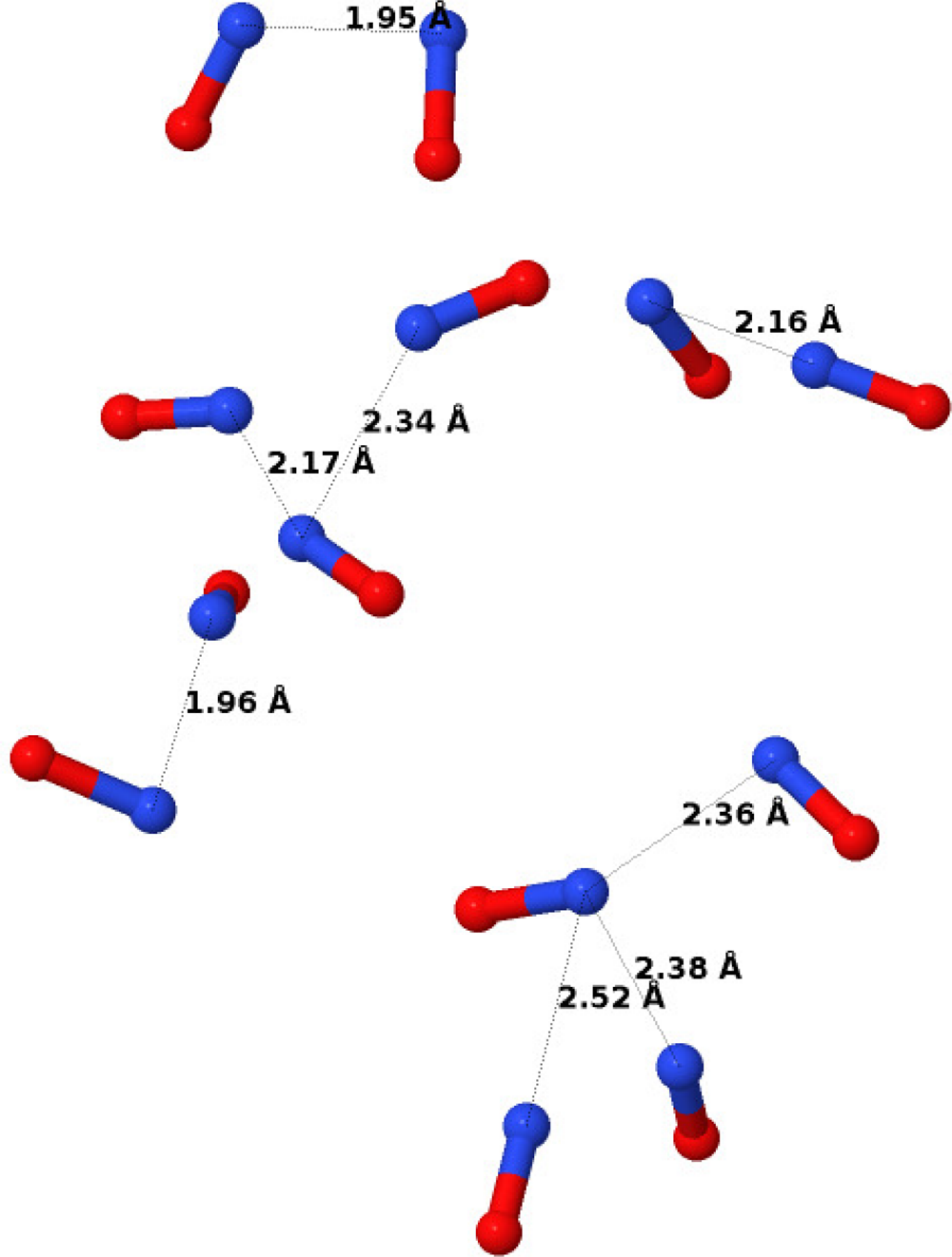
Examples of dimers, trimers, and tetramers found in the simulation of liquid NO at 120 K using only NO monomers, as described in Section 3.2. Single NO molecules have been removed from the display for clarity.

To highlight some of the local geometries found in this second set of simulations, we show in Figure 7 some individual configurations of NO dimers, trimers, and tetramers found in the simulation box at 120 K. It is apparent that there is no *necessity* for the dimers to be planar as described in Section 3.1 and shown in Figure 3: other molecular conformations are also possible without seriously undermining the fit to the data. Furthermore, short chains of three or more NO molecules are possible according to this view—they are not ruled out by the data.

**Figure 8:**
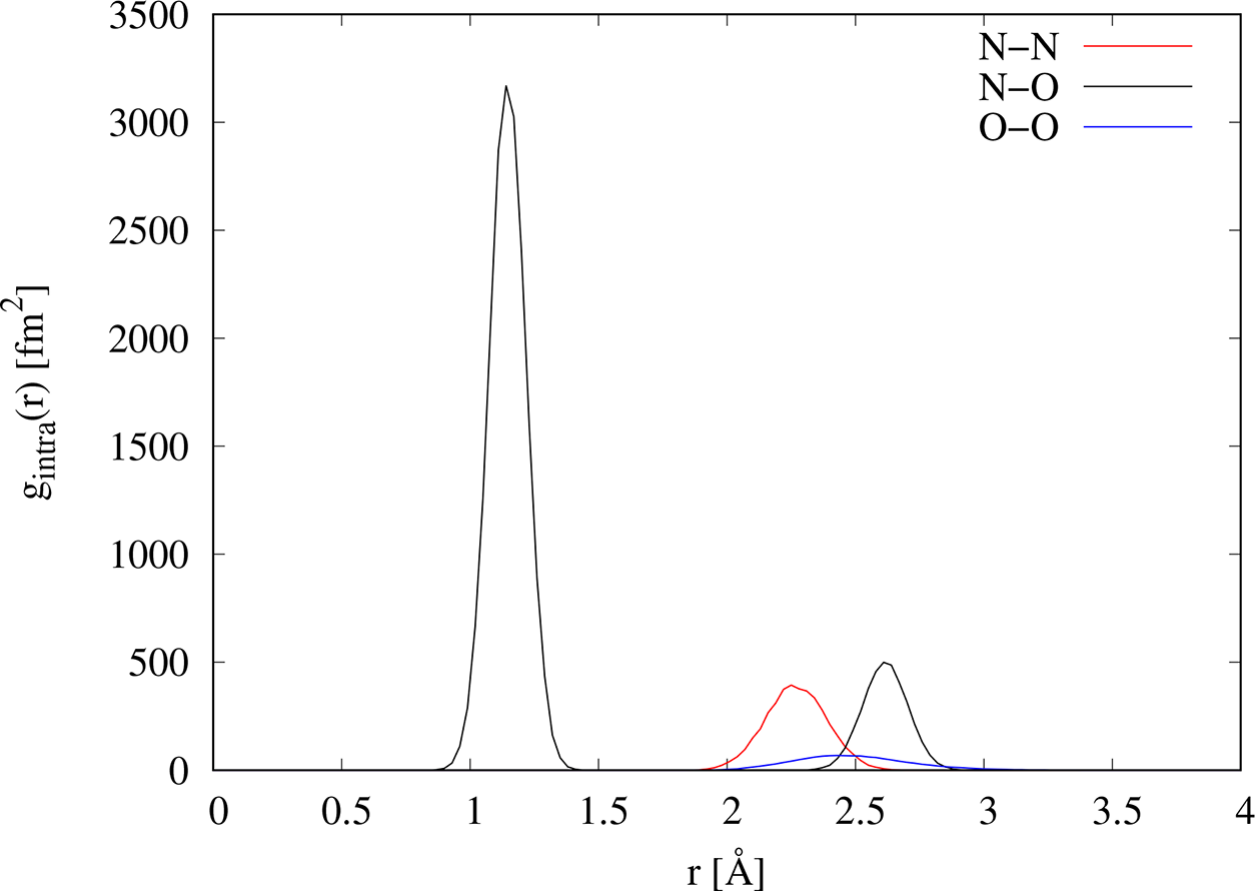
Weighted intramolecular site–site *g*(*r*) for (NO)_2_ molecules. The N–N (red), N–O (black), and O–O (blue) terms have been weighted by the neutron scattering factors 175.2, 217.2, and 67 fm^2^, respectively, corresponding to the stated neutron scattering lengths for N and O.

Several theoretical studies^3,18−20,55,56^ have discussed *ab initio* studies of (NO)_2_ dimers. These studies examine different conformations, some with the oxygen atoms closer to each other than the nitrogen atoms or with a N–N–O–O conformation. On the basis of the present neutron data, we think these other conformations are unlikely in the liquid studied here. The reason is that the neutron scattering length of nitrogen, *b*_N_ = 9.36 fm, is significantly larger than for oxygen, *b*_O_ = 5.80 fm,^57^ and this strongly affects the observed peak heights corresponding to N–N, N–O, and O–O correlations within the diffraction data. These peaks are highlighted in the inset to Figure 4, and the intramolecular site–site *g*(*r*)s, weighted by their corresponding neutron factors , 4*b*_N_*b*_O_, and , are shown in Figure 8. For example, if the O–O distance were shorter than the N–N distance, then the predicted relative height of the two resolvable peaks in Figure 4 would be quite different to what is observed, and an acceptable fit to the diffraction data would not be possible. Therefore, the data give clear evidence that the N–N distance in the dimer, trimer, and so on is shorter than the corresponding N–O and O–O distances within the same oligomers (that is, the N–O and O–O distances between neighboring monomers). The apparently well-defined N–N–O angle in the dimer leads to a correspondingly well-defined N–O second-neighbor distance at about 2.62 Å, which is also seen in the data, because the O–O contribution (Figure 8) to the total is quite weak. However, much less clear is how well-defined is the O–O distance within the dimer. The neutron data shown here appear to conclude that models where this distance is quite well-defined, as in the planar (NO)_2_ dimer (Figure 3), are equally possible as is a looser O–O distance with a much broader range of O–N–N–O dihedral angles. This almost certainly arises in the model because of the weaker scattering power of O compared to N when using neutrons. By the same token, the neutron data do not sharply distinguish between having only (NO)_2_ dimers, or having a range of oligomers, bonded between the nitrogen atoms on each monomer, with relatively unconstrained dihedral angles along the N–N bond.

### 4.2 Mole Fraction of Dimers as a Function of Temperature

**Figure 9:**
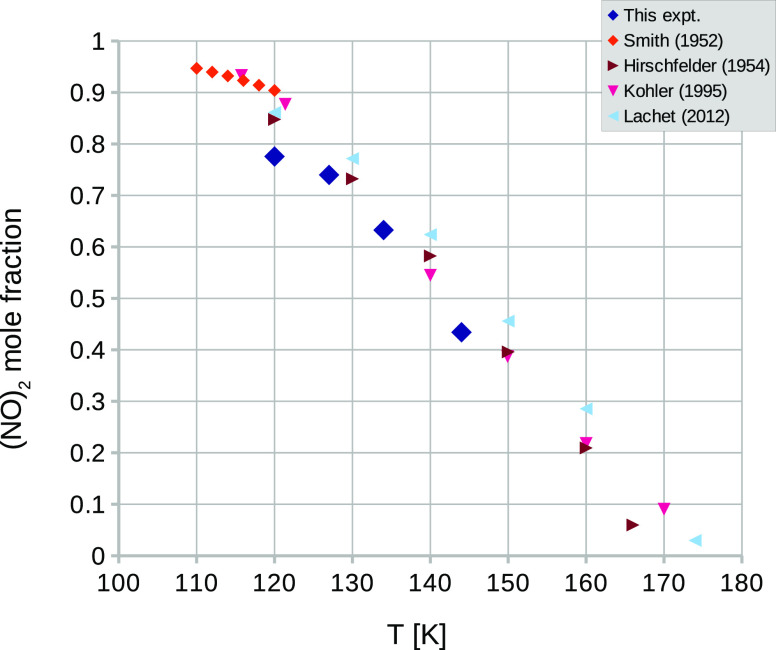
Mole fraction of (NO)_2_ dimers in liquid NO. The present experimental results are taken from Table 5. The results for Hirschfelder (1954) and Lachet (2012) are adapted from Figure 8 of ref (25). The results for Smith (1952) are derived from values of the degree of dissociation given in Table IIIA in ref (21) while the results for Kohler (1995) are taken from Table 7 in ref (15).

In Figure 9 we compare the mole fraction of (NO)_2_ dimers in the liquid as a function of temperature, as obtained in this work (Table 5) with that obtained in several previous experimental and theoretical studies.^15,21,22,25^ In the case of Smith and Johnston,^21^ the quantity measured is the degree of dissociation of NO, namely α. Because it requires two NO monomers to form the dimer, this means the mole fraction of the dimer is (1 – α)/(1 + α).

It can be seen that there is general agreement between the different experiments and theoretical studies, although the present neutron work appears slightly below previous estimates at low temperature. Whether this difference is significant is unknown because it is difficult to put a precise estimate on the uncertainty in these experimental numbers. However, if we assume there is a not-unreasonable uncertainty of 10% on the neutron diffraction numbers, then the discrepancy is not significant. It would be helpful here, following Smith and Johnston,^21^ to have magnetic susceptibility measurements to higher temperatures. However, all the available studies, including the present one, point to almost zero dimer mole fraction at and above the critical point at 180 K.

### 4.3 Comparison with Previous MD Results

**Figure 10:**
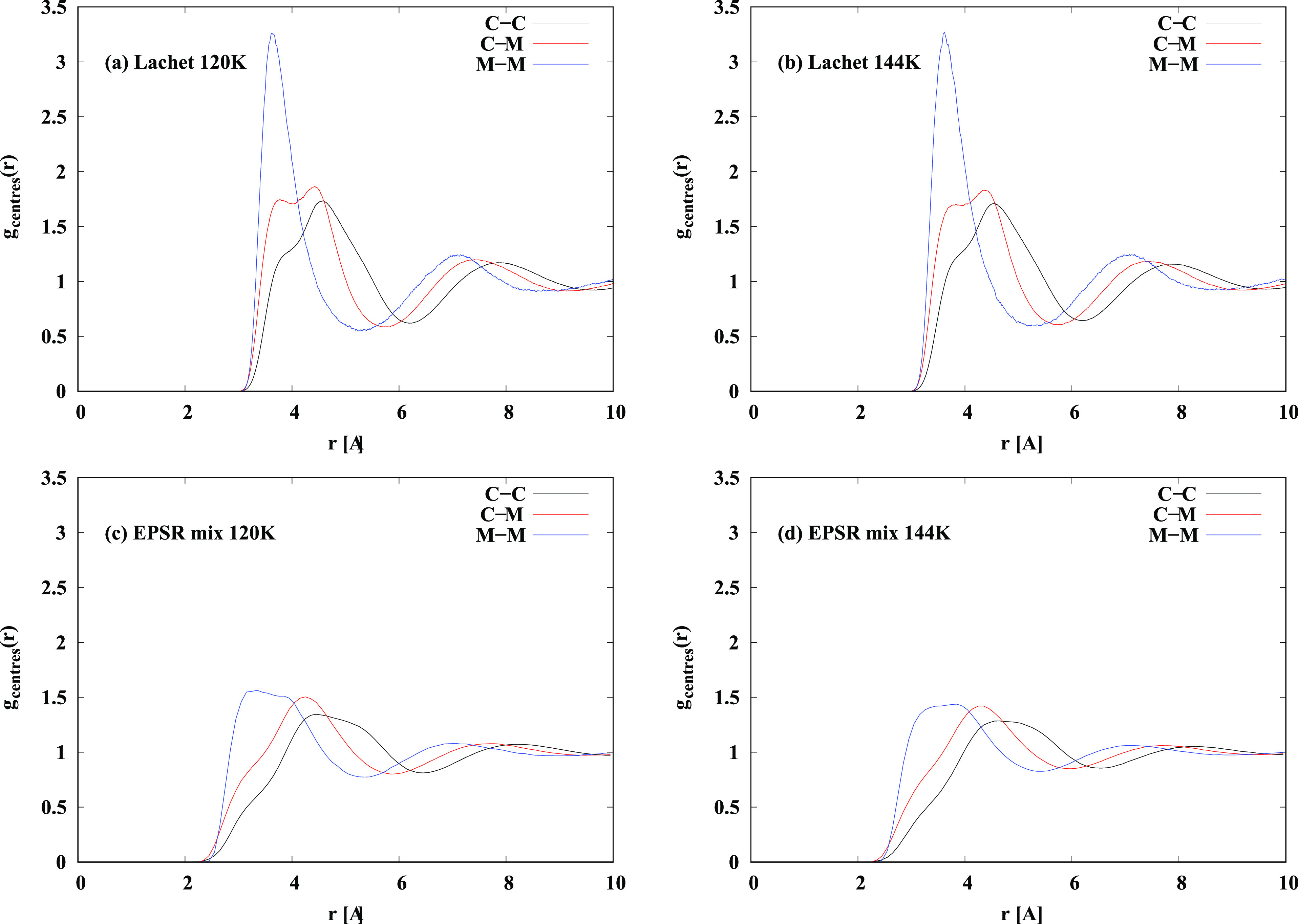
Molecular dynamics simulation of a mixture of (NO)_2_ and NO monomers, based on the potential of Lachet et al.^25^ at two temperatures: 120 K (a) and 144 K(b). The assumed mole fraction of dimers used in the MD is the same as that derived from the current experiment (Figure 9). Also shown, (c) and (d) are the corresponding EPSR simulations at the same temperatures, 120 and 144 K, respectively. C represents the center of mass of the dimer, and M represents the center of mass of the monomer.

Direct comparison of the present EPSR results with previous MD simulations of liquid NO is difficult because the earlier simulations are mostly performed with a simplified model of the NO molecule, and dimer,^22,23,25^ with the NO molecule represented as a single atom and the (NO)_2_ dimer represented as a diatomic molecule. For the present comparison, the Lachet et al. potential was used in an MD simulation and used to calculate the center–center distributions for the dimers, C–C, the center–center distribution for the monomers, M–M, and the center–center distribution for dimer to monomer, C–M. The same can be done for the EPSR simulations described in Section 3.1 by placing a fictitious, noninteracting atom at the centers of mass of each molecule, and the results are shown in the Figure 10.

It can be seen that although there are obvious similarities between MD and EPSR, the lack of the correct atomistic information in the Lachet potential suggests that while this kind of potential may capture the thermodynamics of the system, it will be less accurate at capturing the structure. Clearly more theoretical work on this system is needed.

## 5 Conclusion

The foregoing discussion suggests that nitric oxide is an unusual material in the liquid form. Close to the ambient pressure boiling point the liquid consists of dimers, and possibly trimers, and higher-order oligomers, with the N–N distance between the monomers shorter than either of the corresponding N–O and O–O distances. We assert here that this kind of like–like association between monomers is rare among molecular liquids because it leads to much shorter intermonomer distances than might otherwise be expected based on the van der Waals radii of the corresponding atoms and has some similarities with the phenonmenon of hydrogen bonding in aqueous systems. In both cases a relatively short intermolecular association gives rise to pronounced short-range orientational correlations between the neighboring molecules. The phenomenon is of course much stronger in the case of hydrogen bonding: in the present case the degree of association appears to decrease quite steadily with increasing temperature, dropping from about 0.8 dimers at 120 K to less than 0.5 dimers at 144 K. In the case of water, there is likely some degree of orientational association between neighboring molecules above the liquid–gas critical point,^58^ whereas in NO the presence of dimers above the corresponding critical point for NO is much less likely. We believe this liquid is ripe for a thorough theoretical investigation to characterize this unusual type of monomeric association: existing theoretical work does not deal adequately with the condensed liquid state of nitric oxide. We anticipate reporting on this theoretical work in a future study.

## Notes

The Gudrun and EPSR input and output files can be downloaded from this site: http://purl.org/net/edata/handle/edata/925. The original raw neutron data for this work can be obtained from ref (59).

## Supporting Information Available

Details of the reference potentials used in the EPSR simulations used in this work (PDF)
