# Supplementary material for: Microscopic Structure of Liquid Nitric Oxide
Source: J Phys Chem B. 2022 Nov 18;126(47):9860–70. doi: 10.1021/acs.jpcb.2c05384 (PMC9720726; doi:10.1021/acs.jpcb.2c05384)
Supplement: Supplementary file 1 — jp2c05384_si_001.pdf [file jp2c05384_si_001.pdf]

Supplementary Information for “The Microscopic Structure of Liquid Nitric Oxide”

by Sarantos Marinakis, Cillian Cockrell, Kostya Trachenko, Thomas F. Headen, and Alan K. Soper

Reference potentials used in EPSR simulations of liquid NO

In this note, we summarise the contributions to the Reference Potentials used in the EPSR simulations of liquid NO, in the case where the simulation consists of a mixture of $(NO)_2$ and NO molecules (Section 3.1) and where single NO molecules are “encouraged” to form dimers, and possibly trimers, tetramers, etc., by means of Gaussian potentials, and repulsive exponential potentials, in addition to the standard Lennard-Jones parameters (Section 3.2).

It should be borne in mind that these were designed in the context of EPSR, which is basically a fitting program, and they may or may not have any relevance to the real liquid potentials. In addition EPSR uses molecules that are all slightly different in size (and shape, where relevant) from each other, drawn at random from a distribution of molecule sizes (and shapes) that emulates the observed zero-point energy distributions that are found in the real liquid and in the scattering data. This is slightly analogous, but much simpler, to the path integral method using beads of particles to represent a single atom in quantum simulations. If you apply these potentials to the rigid and identical molecules typically found in classical MD it is likely you may not get the same results because of the difference in molecular geometry between the two simulations.

Lennard-Jones potentials

The form of this potential is standard:-

$$U_{\alpha\beta}(r_{ij}) = 4\epsilon_{\alpha\beta}\left[\left(\frac{\sigma_{\alpha\beta}}{r_{ij}}\right)^{n} - \left(\frac{\sigma_{\alpha\beta}}{r_{ij}}\right)^{6}\right] + \frac{q_\alpha q_\beta}{4\pi\epsilon_0 r_{ij}} \quad \text{(A1)}$$

with

$$\varepsilon_{\alpha\beta} = \sqrt{\varepsilon_\alpha \varepsilon_\beta};\ \sigma_{\alpha\beta} = \frac{1}{2}(\sigma_\alpha + \sigma_\beta) \quad \text{(A2)}$$

For the EPSR simulations discussed in Section 3.1 of the main paper the parameters used are shown in Table 3 of the main paper and repeated here:-

Atom	ε [kJ/mol]	σ [Å]	$q[e]$
N	0.124	3.31	-0.0286
O	0.204	2.95	+0.0286

There were no additional repulsive or Gaussian potentials of the kind described below used for these simulations.

For the EPSR simulations discussed in Section 3.2 of the main paper the parameters used are shown in Table 4 of the main paper and repeated here:-

Atom	ε [kJ/mol]	σ [Å]	$q[e]$
N	0.010	2.90	-0.0286
O	0.010	2.99	+0.0286

Repulsive potentials

These are of the form:-

$$U_{rep}^{(\alpha\beta)}(r)=C_{\min}^{(\alpha\beta)}\exp\left(\Delta_{\min}^{(\alpha\beta)}(r)\right) \quad \text{where} \quad \Delta_{min}^{(\alpha\beta)}(r)=\frac{\left(|r_{min}^{(\alpha\beta)}|-r\right)}{\sigma_{rep}} \quad \text{and} \quad \sigma_{rep}=0.3 \text{ Å.} \tag{A3}$$

In EPSR the coefficients $C_{\min}^{(\alpha\beta)}$ adjust automatically up or down as the empirical potential develops to achieve a specified average coordination number, $N_{min}^{(\alpha\beta)}$, out to the specified distance $|r_{min}^{(\alpha\beta)}|$, so we give only typical values for these coefficients. If $r_{min}^{(\alpha\beta)}<0$ then $C_{\min}^{(\alpha\beta)}$ is fixed at the stated value, but there is no control of the coordination number in that case. Note that $C_{\min}^{(\alpha\beta)}$ can go negative if required to satisfy a particular coordination number and depending on what the EP is doing, making the “repulsive” potential actually attractive in that case. If $r_{min}^{(\alpha\beta)}$ is zero then the corresponding coefficient, $C_{\min}^{(\alpha\beta)}$, is automatically set to zero and there is no repulsive potential in that case. If the specified coordination number is zero the coefficient $C_{\min}^{(\alpha\beta)}$ will never go below zero.

For N-N, N-O and O-O the values of $r_{min}^{(\alpha\beta)}$ are 2.55, 2.85, and 0.00 Å respectively. The values of $N_{min}^{(\alpha\beta)}$ and typical values of $C_{\min}^{(\alpha\beta)}$ are given in the following table:-

T [K]	$N_{min}^{(NN)}$	$C_{\min}^{(NN)}$ [kJ/mole]	$N_{min}^{(NO)}$	$C_{\min}^{(NO)}$ [kJ/mole]	$N_{min}^{(OO)}$	$C_{\min}^{(OO)}$ [kJ/mole]
120	0.80	-0.25708	1.00	0.56357	0.00	0.00
127	0.76	1.32931	0.80	1.57191	0.00	0.00
134	0.65	1.17805	0.80	1.42600	0.00	0.00
144	0.45	0.79024	0.80	0.64451	0.00	0.00

Additional Gaussian potentials

In addition to the terms already described it is possible to add up to 5 Gaussian contributions to the Reference Potential to simulate bonding interactions between atoms. These potentials have the form

$$U_{Gauss}^{(j)}(r)=\sum_{i=1}^{5} h_i^{(j)}\exp\left[-\frac{1}{2}\left(\frac{p_i^{(j)}-r}{w_i^{(j)}}\right)^2\right] \tag{A4}$$

where $p_i^{(j)}$, $w_i^{(j)}$ and $h_i^{(j)}$ are the position, width and height of the ith term of the jth atom pair respectively. There was one such term for the NN atom pair and two terms for the NO and OO atom pairs:-

j	$p_1^{(j)}$ [Å]	$w_1^{(j)}$ [Å]	$h_1^{(j)}$ kJ/mole	$p_2^{(j)}$ [Å]	$w_2^{(j)}$ [Å]	$h_2^{(j)}$ kJ/mole
N-N	2.26	0.10	-1.3	-	-	-
N-O	2.61	0.10	-0.8	2.9	0.1	1.0
O-O	2.44	0.15	-0.4	2.9	0.1	1.0

The reference potentials that are generated by these parameters are shown in Fig. 6 of the main paper.

Truncation of potentials.

The non-Coulomb part of the potentials, including the added Gaussian terms, are truncated by a function of the form

$$T(r) = \begin{cases} 1 & r<r_{minpt} \\ 0.5\left[1+\cos\pi\left(\frac{r-r_{minpt}}{r_{maxpt}-r_{minpt}}\right)\right] & r_{minpt}<r<r_{maxpt} \\ 0 & r>r_{maxpt} \end{cases} \quad \text{(A5)}$$

with r_{minpt} = 9 Å and r_{maxpt} = 12 Å.

For the Coulomb term in (A1) the “charged clouds” truncation function takes the form originally proposed by Hummer *et al.*, *J. Phys. Condens. Matter,* **6**, A141 (1994):

$$T_C(r)=\left(1-\frac{r}{r_{maxpt}}\right)^4\left(1+\frac{8r}{5r_{maxpt}}+\frac{2r^2}{5r_{maxpt}^2}\right)\Theta\left(r_{maxpt}-r\right) \quad \text{(A6)}$$

with $\Theta\left(r_{maxpt}-r\right)$ the Heaviside function.
